# Chilaiditi syndrome presenting as chest pain in an adult patient: a case report

**DOI:** 10.1186/1752-1947-8-97

**Published:** 2014-03-16

**Authors:** Ying-Yi Chen, Hung Chang, Shih-Chun Lee, Tsai-Wang Huang

**Affiliations:** 1 Division of Thoracic Surgery, Tri-Service General Hospital, National Defense Medical Center, Taipei, Taiwan

**Keywords:** Chilaiditi syndrome, Pneumoperitoneum, Subdiaphragmatic colon

## Abstract

**Introduction:**

A patient with chest contusion and rib fractures presented with severe chest pain. The plain film of his chest showed suspicion of pneumoperitoneum. We present this case to show how to get a correct diagnosis and then avoid unnecessary surgery.

**Case presentation:**

A 64-year-old Taiwanese man presented to the emergency department complaining of severe right chest pain after a traffic accident. Chest radiography showed right fifth to eighth rib fractures and was suspicious for free air under the bilateral hemi-diaphragm. Computed tomography of the abdomen revealed interposition of bowel loops between the liver and diaphragm. The patient was treated with oral analgesics and then regularly followed in the outpatient department.

**Conclusion:**

Awareness of Chilaiditi’s sign is of paramount importance when free air under the diaphragm is seen in a patient (particularly an older patient) who does not exhibit signs of peritoneal irritation on physical examination. Emergent laparotomy should be delayed and a computed tomography scan should be done first. No inappropriate surgical intervention is needed.

## Introduction

We present the case of a patient with chest contusion, with rib fractures and severe chest pain. The plain film of our patient’s chest showed free air at his bilateral subdiaphragm, suspicious of pneumoperitoneum. Before we considered emergent surgical intervention, we got a strong indication of what would benefit our patient. Therefore, we report our patient as showing unusual symptoms, and we show how we made a correct diagnosis. For thoracic trauma patients, a computed tomography (CT) scan can differentiate whether the air is free or intraluminal and show how we can avoid inexpedient surgical intervention, including laparoscopy or laparotomy.

## Case presentation

**Figure 1:**
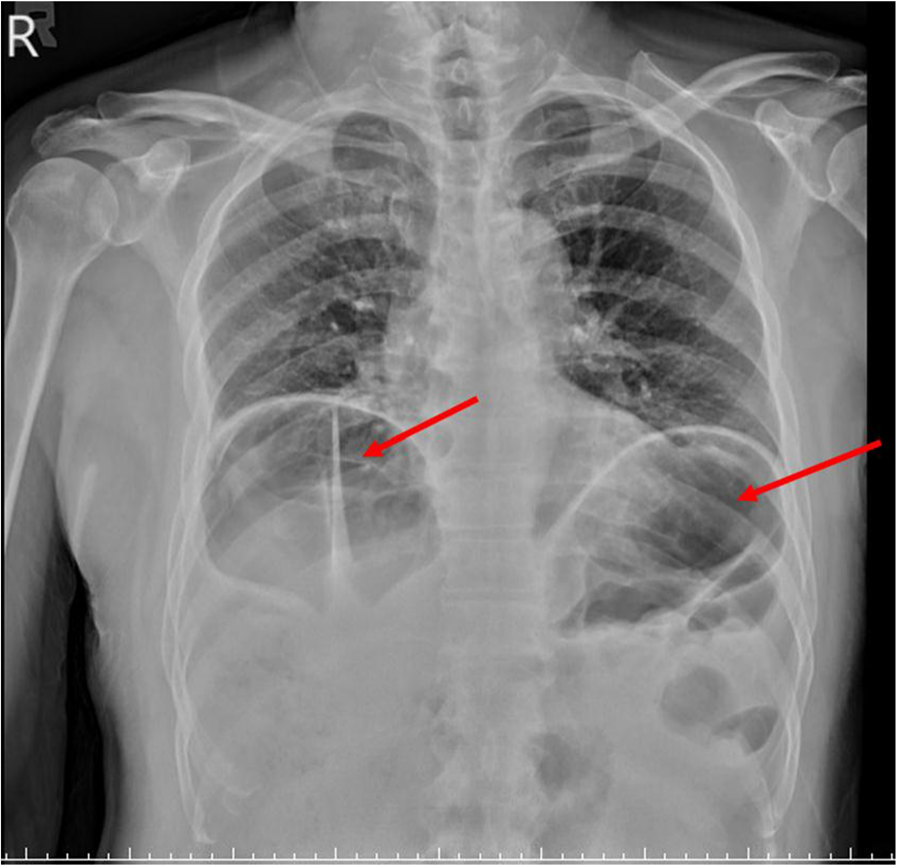
**Plain film of chest.** Chest radiography showed right fifth to eighth rib fractures and was suspicious for free air under our patient’s bilateral hemi-diaphragm (red arrows).

**Figure 2:**
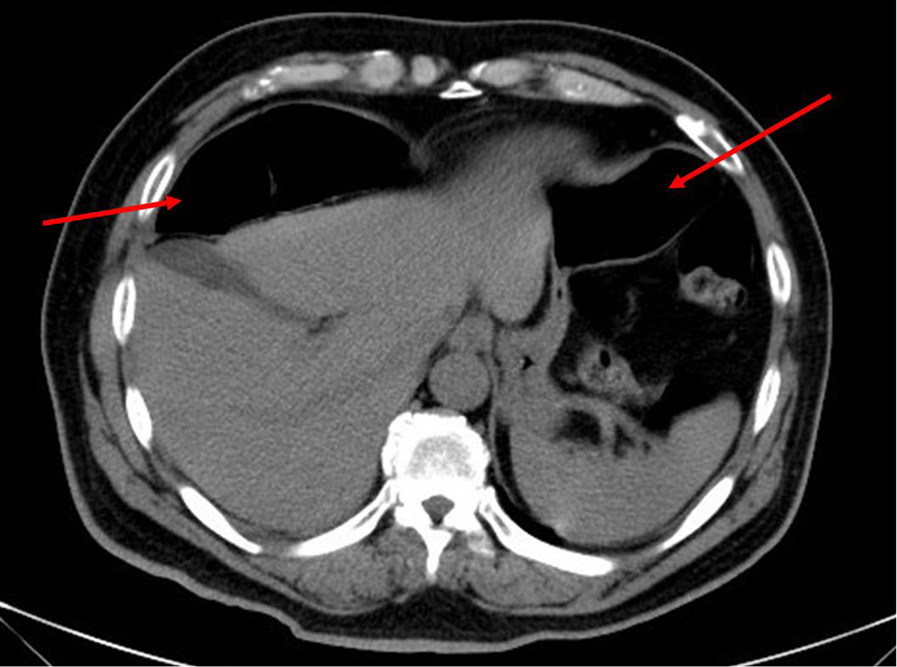
**Computed tomography of the abdomen.** Red arrows show the interposition of bowel loops between our patient’s liver and diaphragm.

A 64-year-old man presented to our emergency department complaining of right chest pain after a traffic accident. He denied having any other systemic diseases. On physical examination, he was afebrile (temperature 36.9°C) with normal vital signs (blood pressure 131/91mmHg, heart rate 77bpm). Pulse oximetry revealed 98% saturation on room air. His chest was clear bilaterally with a midline trachea and no crepitus, but with substantial right-sided posterolateral chest wall tenderness with decreased breathing sounds. The abdomen was soft, non-tender and non-distended, with no palpable intra-abdominal masses or organomegaly. Rectal examination was negative for gross blood or masses. His extremity examination was unremarkable. Chest radiography showed right fifth to eighth rib fractures and was suspicious for free air under his bilateral hemi-diaphragm (Figure 1). CT of the abdomen revealed interposition of bowel loops between the liver and diaphragm (Figure 2). He was treated with oral analgesics and discharged to his home five days later. During a six-month follow-up period, his recovery was uneventful.

## Discussion

Chilaiditi’s sign, or hepatodiaphragmatic interposition, is an important part of the differential diagnosis in patients with free air under the diaphragm. In 1910, a Viennese radiologist, Demetrius Chilaiditi, described three cases of hepatodiaphragmatic interposition, and termed the condition “hepatoptosis” [1]. This radiologic finding later became known as Chilaiditi’s sign and is increasingly recognized as an important imitator of free air under the diaphragm. Chilaiditi’s sign is usually an incidental radiologic finding in a patient who is asymptomatic [2]. When associated with vague abdominal pain, distension, vomiting, anorexia and constipation, this entity is termed Chilaiditi’s syndrome [3]. Chilaiditi’s sign has a reported incidence of 0.025% to 0.28% as diagnosed by upright chest radiographs [4] in the general population. It is four times more common in male patients than in female patients [2], and is much more common in older people (1% in a retrospective study of a geriatric population) [5].

Hepatodiaphragmatic interposition is inhibited by normal upper abdominal anatomy and is predisposed in any condition that augments the hepatodiaphragmatic recess. These conditions include ascites or high abdominal fat content, decreased liver volume (owing to cirrhosis or resection), as well as weakness or division of the suspensory ligaments. In addition, patients with megacolon or a long, redundant colon with a long, narrow mesentery are at increased risk. Differentiation of this finding from true pneumoperitoneum (or subdiaphragmatic abscess, another item on the differential diagnosis of free air under the diaphragm) is critically important. As in our case, a CT scan of the abdomen can be extremely helpful in differentiating among these three conditions. Alternatively, lateral decubitus films may be useful, since in the case of hepatodiaphragmatic interposition or subdiaphragmatic abscess, unlike pneumoperitoneum, the “free air” will remain in the hepatodiaphragmatic recess and not lay to the independent paracolic gutter.

In another case report, Tangri Nitin *et al.*[6] reported a 50-year-old man with combined Chilaiditi sign and pneumothorax. It is important to consider the possibility of inadvertent bowel injury while managing pneumothorax. Further investigation may help in understanding the etiopathogenesis of both conditions, as a true etiology remains unclear. However, our patient complained of left-sided chest pain, which was easy to differentiate from the right-sided Chilaiditi sign. Compared with the symptoms of our patient, physicians of this older man, who had right-sided trauma with chest pain, were liable to make a diagnostic error.

In the evaluation of an injured patient, pneumoperitoneum on the initial chest radiograph is typically an indication for immediate laparotomy. Often, further imaging studies are omitted for fear of delay and on-going abdominal contamination. Chest contusion with rib fractures should increase suspicion of abdominal injuries. For thoracic trauma patients, a CT scan can differentiate whether the air is free or intra-luminal and an inexpedient laparotomy or laparoscopy can be avoided.

## Conclusions

Awareness of Chilaiditi’s sign is of paramount importance when free air under the diaphragm is seen in a patient (particularly an older patient) who does not exhibit signs of peritoneal irritation on physical examination. Emergent laparotomy should be delayed and a CT scan should be done first. No inappropriate surgical intervention is needed.

## Consent statement

Written informed consent was obtained from the patient for publication of this case report and any accompanying images. A copy of the written consent is available for review by the Editor-in-Chief of this journal.

## Abbreviations

CT: Computed tomography.

## Competing interests

The authors declare that they have no competing interests.

## Authors’ contributions

YY analyzed and interpreted the patient data regarding the medical record and organized the history of the present illness. HC and SC helped to collect patient data and review the article. TW was a major contributor in writing the manuscript. All authors read and approved the final manuscript.
